# Daily Rhythms of the Expression of Key Genes Involved in Steroidogenesis and Gonadal Function in Zebrafish

**DOI:** 10.1371/journal.pone.0157716

**Published:** 2016-06-20

**Authors:** Viviana Di Rosa, Jose Fernando López-Olmeda, Ana Burguillo, Elena Frigato, Cristiano Bertolucci, Francesc Piferrer, Francisco Javier Sánchez-Vázquez

**Affiliations:** 1 Department of Physiology, Faculty of Biology, Regional Campus of International Excellence “Campus Mare Nostrum”, University of Murcia, Murcia, Spain; 2 Department of Life Sciences and Biotechnology, University of Ferrara, Ferrara, Italy; 3 Institut de Ciències del Mar, Consejo Superior de Investigaciones Científicas (CSIC), Barcelona, Spain; Karlsruhe Institute of Technology, GERMANY

## Abstract

Fish present daily and seasonal rhythms in spawning and plasmatic levels of steroids that control reproduction. However, the existence of the rhythms of expression of the genes that underlie the endocrine mechanisms responsible for processes such as steroidogenesis and reproduction in fish have still been poorly explored to date. Here we investigated the daily pattern of the expression of key genes involved in sex steroid production that ultimately set the sex ratio in fish. Adult zebrafish were maintained under a 12:12 h light-dark cycle at a constant temperature of 27°C and were sampled every 4 h during a 24-hour cycle. The expression of key genes in the gonads and brains of female and male individuals were analyzed. In gonads, the expression of aromatase (*cyp19a1a*, ovarian aromatase) and the antimüllerian hormone (*amh*, testis) was rhythmic, with almost opposite acrophases: ZT 5:13 h (in the light phase) and ZT 15:39 h (at night), respectively. The expression of *foxl2* (forkhead box L2) was also rhythmic in the ovary (acrophase located at ZT 5:02 h) and the expression of *dmrt1* (doublesex and mab-3-related transcription factor 1) was rhythmic in testes (acrophase at ZT 18:36 h). In the brain, *cyp19a1b* (brain aromatase) and *cyp11b* (11beta-hydroxylase) presented daily differences, especially in males, where the expression peaked at night. These results provide the first evidence for marked time-of-the-day-dependent differences in the expression of the genes involved in sex ratio control, which should be considered when investigating processes such as reproduction, sex differentiation and steroidogenesis in fish.

## Introduction

Animals that live according to environmental cycles have developed rhythmic physiological and behavioral processes that are driven by an internal time-keeping system. Biological clocks allow animals to anticipate these cyclic events (e.g., day/night or season) and to cope with them better. Alternation of light and darkness (the 24-hour LD cycle) represents the main time cues capable of entraining biological clocks, and allows the phase of endogenous rhythms to be set with the phase of cyclic events in the environment [1]. In ectothermic animals, such as fish, temperature cycles play a relevant role as the water dynamics creates an ecosystem in which animals have to adapt to live [2–3].

Fish reproduction exhibits both daily and seasonal rhythms in most species. A number of investigations have reported daily variations in sex steroids, such as estradiol (E2), testosterone, 11-ketotestosterone (11-KT) and progesterone, in different fish species: Japanese charr (*Salvelinus leucomaenis*) [4], wrasse (*Pseudolabrus sieboldii*) [5], catfish (*Heteropneustes fossilis*) [6] and Senegalese sole (*Solea senegalensis*) [7]. Daily changes in oocyte maturation and secretion of sex steroids have also been reported in bamboo leaf wrasse (*Pseudolabrus japonicas*) [8], snapper (*Pagrus auratus*) [9], kisu (*Sillago japonica*) [10] and gilthead sea bream (*Sparus aurata*) [11]. In accordance with fish seasonal reproduction, annual changes in sex steroids have been reported in European sea bass (*Dicentrarchus labrax*) [12–13], rainbow trout (*Oncorhynchus mykiss*) [14] and Senegalese sole [7]. Previous research has also highlighted the effect of photoperiod and temperature as having a potential influence on fish sex ratios [15–23]. Daily cycles of environmental factors have been reported to act differently during sex differentiation; e.g. thermo-cycles induce a high proportion of females in zebrafish, whereas constant temperature leads to more males [24].

Zebrafish is mostly a diurnal species [25], but is capable of displaying either diurnal or nocturnal behavioral rhythms (i.e., nocturnal self-feeding) [26]. Daily thermocycles can also drive behavioral rhythms in zebrafish [27–28]. When this species is submitted to a long photoperiod (LD 14:10 h), fish spawn at the beginning of the light phase. The diurnal spawning rhythm is maintained in the light phase, even when zebrafish are fed at night, and despite their locomotor activity becoming nocturnal. Zebrafish’s ability to sustain a diurnal spawning rhythm confirms the strong influence of the LD-cycle on the entrainment of spawning rhythms [29]. Daily variations in *fsh* (follicle-stimulating hormone) and *lh* (luteinizing hormone) production in the zebrafish pituitary have been reported, which could be related with the daily rhythms of zebrafish spawning [30]. However, nothing is known about rhythmicity in other factors involved in sex steroid production in this species, especially at the gonadal level.

Timing of reproduction and production of sex steroids is controlled by the hypothalamic-pituitary-gonad axis (BPGa), which is necessary for multiple processes like sex differentiation, gonad maturation and spawning [11, 31]. Aromatase plays a key role during sex differentiation and gonad maturation, and in most fish it presents two different genes: *cyp19a1a* and *cyp19a1b*. The former is also called “ovarian aromatase” as it is expressed mainly in differentiating and adult gonads of teleost fish. It is an enzymatic complex that facilitates estrogen synthesis from testosterone or androstenedione [32]. *Cyp19a1b is* also called “brain aromatase” because this gene is highly expressed in the teleost brain of both females and males [33]. In fish, *cyp19a1a* is essentially expressed only in gonads, while *cyp19a1b* is expressed mostly in the brain, but can also be found in other tissues [34]. In testes, the antimüllerian hormone (*amh*) initiates the regression of Müllerian ducts and inhibits the expression of aromatase (*cyp19a1a*) to avoid the transformation of androgens into estrogens. *Foxl2* (forkhead box L2) is a transcription factor that is known as a potent transcriptional activator of *cyp19a1a* [35]. Thus *foxl2* plays an important role in those species in which temperature affects the sex ratio [36]. High temperature during the thermosensitive period suppresses *cyp19a1a* gene expression to result in low aromatase activity and E2 levels [37]. *Dmrt1* (doublesex and mab-3 related transcription factor 1) has been found expressed in Japanese medaka (*Oryzias latipes*), is an inhibitor of germ cell proliferation, and is expressed only during testicular differentiation. In Nile tilapia (*Oreochromis niloticus*), this factor down-regulates *cyp19a1a* during testicular differentiation [38]. In zebrafish, *dmrt1* is not only associated with testis development, but may be important in ovary differentiation [39]. The *cyp11b* (11beta-hydroxylase) gene contributes to the synthesis of 11-KT from testosterone, which is the most potent androgen in teleost fish, with a higher expression in male gonads than in females [40]. Although rhythms at plasmatic levels of sex steroids and fish reproduction have been described in many species, the rhythmic nature of these key enzymes involved in sex steroidogenesis remains unknown to date.

The aim of this research was to investigate, for the first time, the existence of daily expression patterns of six specific genes that play a key role in fish reproduction and steroidogenesis by using zebrafish as the most appropriate experimental model. These genes were analyzed in two different tissues, gonads and brain, from both sexes. In the ovary we looked at the expression of *cyp19a1a* and *foxl2*, whereas in testes we examined the expression of *amh*, *dmrt1* and *cyp11b*. In the brain of both males and females we analyzed the expression of *cyp19a1b* and *cyp11b*.

## Materials and Methods

### Ethics Statement

The present research was carried out in the Chronobiology laboratories at the University of Murcia (Spain). All husbandry and experimental procedures complied with European Legislation for the Protection of Animals used for Scientific Purposes (Directive 2010/63/EU). The experimental protocol was previously authorized by the Spanish National Committee on Animal Welfare (RD 1201/2005 and law 32/2007) and the Bioethical Committee of the University of Murcia (Spain).

### Animal rearing

Adult wild-type zebrafish (*Danio rerio*) of mixed sexes were obtained from a local provider (Jumipez S.A., Murcia, Spain). Approximate fish body weight was 0.75 g, with a total length of 4 cm. Fish were housed for 6 months in our laboratories. Four weeks before sample collection, fish were divided (N = 160) into two 60-liter aquaria (60x30x32 cm) according to sex, with males placed in one aquarium and females in the other. Each aquarium had a closed water circulation system provided with aeration, and also with mechanical and biological filters. Aquaria were kept in a chronolab, a completely isolated room, where light and temperature were strictly controlled. Temperature was recorded by an underwater data logger (HOBO PENDANT^®^ Onset Computer Corporation, Massachusetts, USA) and was maintained at 27±0.5°C using a water heater (100 W, Askoll, Italy). Lighting conditions were set according to a 12:12 h LD cycle, with light onset at 8 am (*Zeitgeber* Time 0 h, ZT 0 h). Fish were fed by an automatic feeder (Eheim, Germany) located in the upper part of the aquaria, which released food (Tropical fish flakes, Prodac, Italy) at a quantity daily rate of 1% of total biomass in each aquarium once a day at ZT 4 h. Each aquarium was equipped with an infrared photocell connected to a computer to record locomotor activity. The photocell was located in the middle of the large side of each aquarium to detect the movement of fish when they interrupted the infrared beam. Interruptions were recorded as signals and were stored in the computer every 10 minutes.

### Sampling and analysis

Samples were collected throughout a 24-hour cycle at six different time points (ZT 2, 6, 10, 14, 18 and 22 h). At each time point, 10 fish (five of each sex) were anesthetized with eugenol (clove oil essence, Guinama, Valencia, Spain) at a concentration of 50 μL/L and sacrificed by decapitation. Fish manipulation and tissue collection in the dark phase were performed under a dim red light. Brains and gonads (ovaries from females, testes from males) were extracted from each fish. Total RNA was isolated from each sample using Trizol reagent (Invitrogen, Carlsbad, CA, USA) following the manufacturer’s instructions. The amount, quality and composition of isolated RNA were analyzed by Nanodrop ND-1000 (Thermo Fisher Scientific Inc., Wilmington, USA). Total RNA (1 μg) was incubated with DNase I (Invitrogen) at room temperature for 30 min and then at 85°C for 15 min to inactivate the enzyme. DNase-treated RNA was used to perform cDNA synthesis in a final volume of 20 μl using the QuantiTect Reverse Transcription Kit (Qiagen, USA). The reaction was performed at 42°C for 30 min, followed by a 5-min inactivation step at 85°C. Then cDNA was PCR-amplified with the StepOnePlus Real-Time PCR System (Applied Biosystems, Foster City, CA, USA) using the SYBR-green primer master mix according to the manufacturer’s recommendations (Applied Biosystems, Foster City, CA, USA). The thermal cycling conditions were as follows: 15 min of denaturation at 95°C, followed by 40 cycles of a 15-s denaturation step at 95°C, and then by an annealing-elongation step for 30 s at 60°C. After amplification, a melting curve analysis was performed to confirm amplicon specificity. All the samples were run in triplicate. The gene-specific primers for *cyp19a1a*, *cyp19a1b*, *cyp11b*, *dmrt1* and *foxl2* were designed with the primer Express software (Applied Biosystems) (Table 1). The primer sequences for *amh* were retrieved from the literature [41]. Efficiency of primers was verified by constructing standard curves for all the investigated genes. The dissociation curve was used to confirm amplicon specificity. The relative expression levels of each sample were calculated by the 2^–ΔΔCT^ method [42]. As housekeeping genes, *βactin* was used in the gonad samples and *loopern4* was used in the brain samples [43]. Housekeeping genes were selected after checking that the coefficient of variation (C.V.) for each gene within each tissue was lower than 5%. The second normalization in the 2^–ΔΔCT^ calculations was performed using the sample with the lowest value within each gene and tissue as the reference.

**Table 1:** Primer sequences used for the quantitative PCR analyses.

Gene	Direction	Primer sequence 5'-3'	Accession number
*cyp19a1a*	F	TGCTGGCCATCAGACACCAT	AF183906
	R	CAGATGAACCGACAGTAGGAGACAA	
*cyp19a1b*	F	TCGGCACGGCGTGCAACTAC	AY780257
	R	CATACCTATGCATTGCAGACC	
*amh*	F	GGGTGTGCATGCTACAGAAGAT	AY677080
	R	CTCAGAAATGCAAACAGTCTGTGT	
*cyp11b*	F	CCTCGGGCCCATATACAGAGA	NM_001080204
	R	CGTCCCGTTCTTGAGGAAGA	
*dmrt1*	F	ATGGCAGAGCAGAACGATTT	NM_205628
	R	TAGTCCCACAACAGCATGGA	
*foxl2*	F	AAACACTGGGAAGGTTTGCGTGC	NM_001045252
	R	TTTGTCCGGCCCCTTCTCTGG	

### Statistical analysis

All the results were expressed as mean±SEM. The significance threshold (α) was set at 0.05 in all the statistical tests. The gene expression data were first subjected to the D'Agostino-Pearson normality test and Bartlett's test of homocedasticity. All the data fulfilled the assumptions of normality of distributions (D'Agostino-Pearson test, p>0.05) and homogeneity of variances (Bartlett's test, p>0.05). Then the data from each gene were subjected to a one-way analysis of variance (ANOVA) to determine of the existence of statistically significant differences between time points. The values from each gene and tissue were subjected separately to a one-way ANOVA. After checking with the one-way ANOVA that the data showed significant differences (p<0.05), a Tukey´s HSD *post hoc* test was used for multiple comparisons between groups (time points). D'Agostino-Pearson tests, Bartlett's test and one-way ANOVAs were performed with SPSS 15.0 (SPSS Inc., Chicago, IL, USA).

The Cosinor analysis was run with the El Temps software (v. 275, Prof. A. Díez-Noguera, University of Barcelona, Spain) to determine whether the daily expression of the genes fitted a cosine function: Y = M+A x[cos (Ωt+Φ)], where M is mesor, A is amplitude, Ω is angular frequency (360°/24 h for the circadian rhythms) and Φ is acrophase. A cosine function was selected because it is the simplest mathematical model that explains the rhythmic process and that which avoids prejudging the correlation/equation to a higher extent. The Cosinor analysis provides a fit value (%V) that indicates the percentage of the variance of the experimental data explained by the cosine equation. This %V is the equivalent to the degree of fitness to the calculated cosine function of the experimental data. The Cosinor analysis also provided the statistical significance of the rhythm through an F-test of the variance accounted for by the waveform versus a straight line of zero-amplitude (null hypothesis). Therefore, if under a statistical significance of p<0.05 this null hypothesis was rejected, amplitude could be considered as differing from 0, thereby constituting evidence for the existence of a statistically significant rhythm of the given period under study. The locomotor activity data were analyzed, and actograms and meanwaves were plotted with El Temps.

## Results

**Fig 1:**
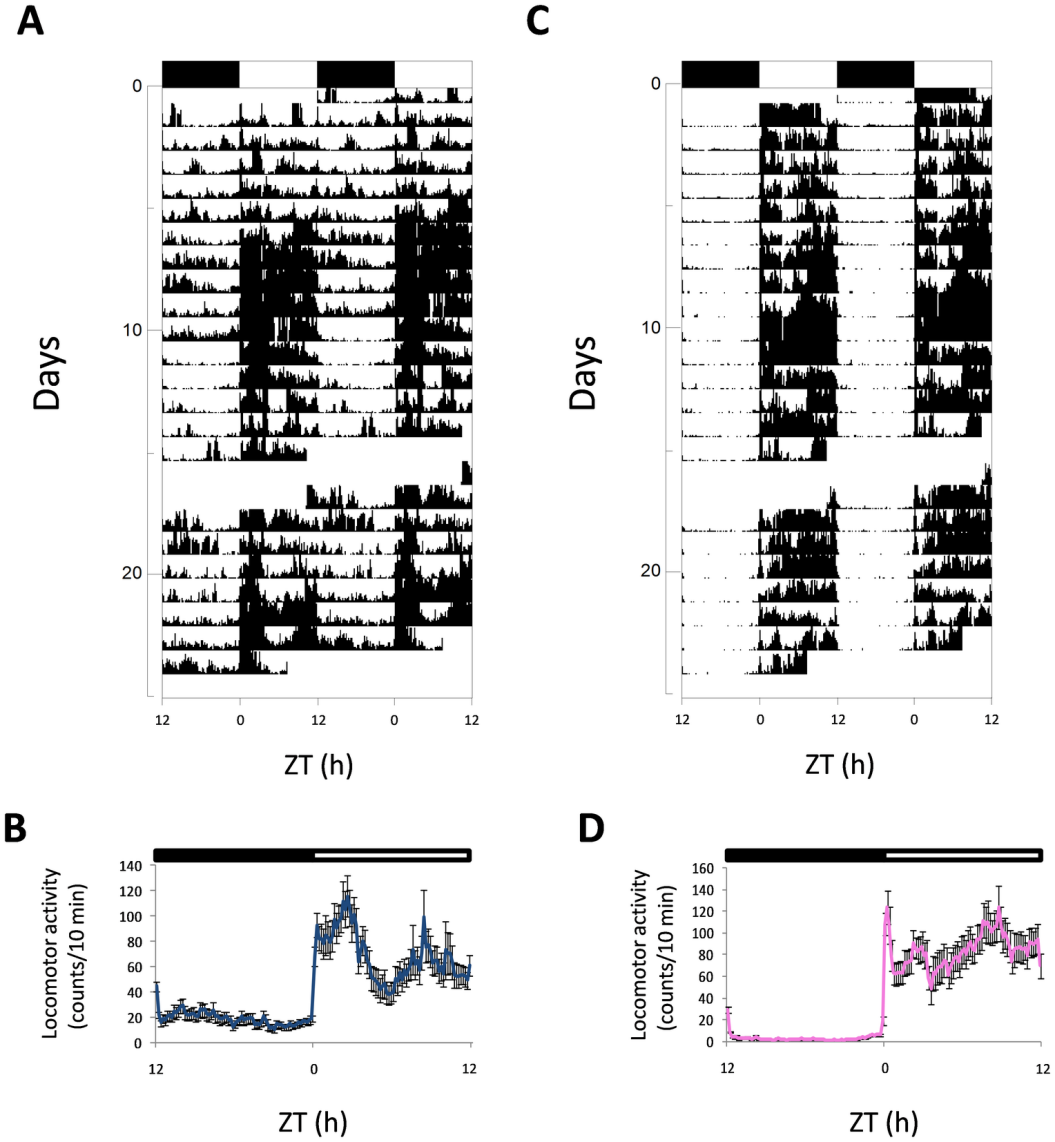
Representative actograms and mean waveforms of locomotor activity of zebrafish males (A, B) and females (C, D). Zebrafish were subjected to 12:12 LD and fed daily at ZT 4 h. Actograms were double-plotted (time scale 48 h). The height of each point represents the number of infrared light-beam interruptions/10 min. Each horizontal line shows one experimental day on the vertical axis, and the hours of the day are represented on the X-axis. The black bar at the top of each actogram represents the dark phase, and the white one represents the light phase of the LD conditions. In the meanwaves, each point was calculated as the mean±S.D from the 10-minute binned data across all the experimental days. Each waveform is represented as single-plotted.

All the male and female zebrafish displayed a diurnal rhythm of locomotor activity and exhibited most of their activity in the light phase, as revealed in the actograms (Fig 1A and 1C for male and female, respectively). Activity increased sharply after lights were switched on, with a high level maintained in the light phase (Fig 1B and 1D).

**Fig 2:**
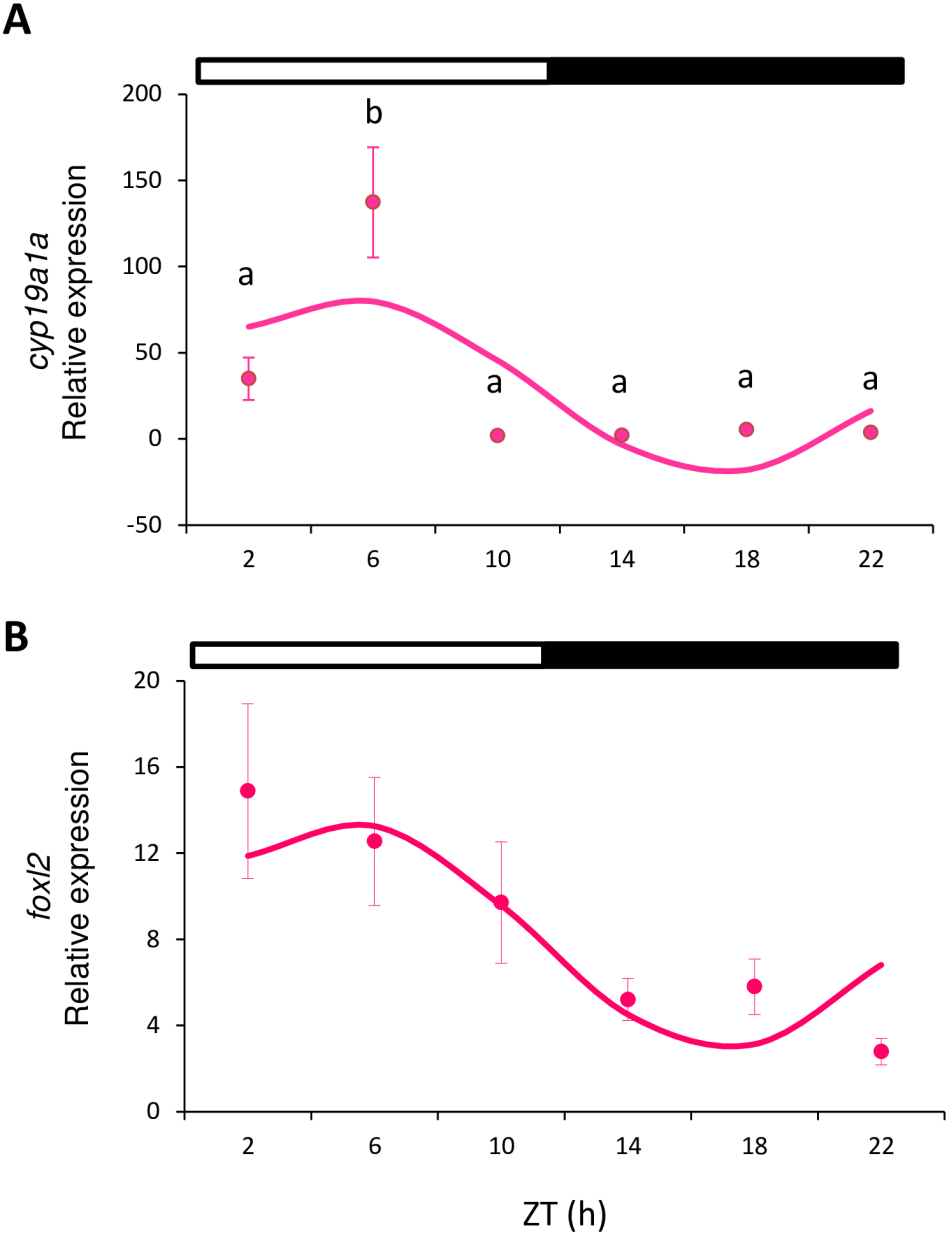
Relative gene expression of *cyp19a1a* (A) and *foxl2* (B) in ovary. Circles indicate the relative gene expression (mean±SEM) at each sampled time point. Different letters indicate the statistically significant differences between time points (one-way ANOVA, p<0.05). The sinusoidal curve calculated by Cosinor (p<0.05) is indicated by the continuous line. The white and black bars above the graphs represent the light and dark phase, respectively.

**Fig 3:**
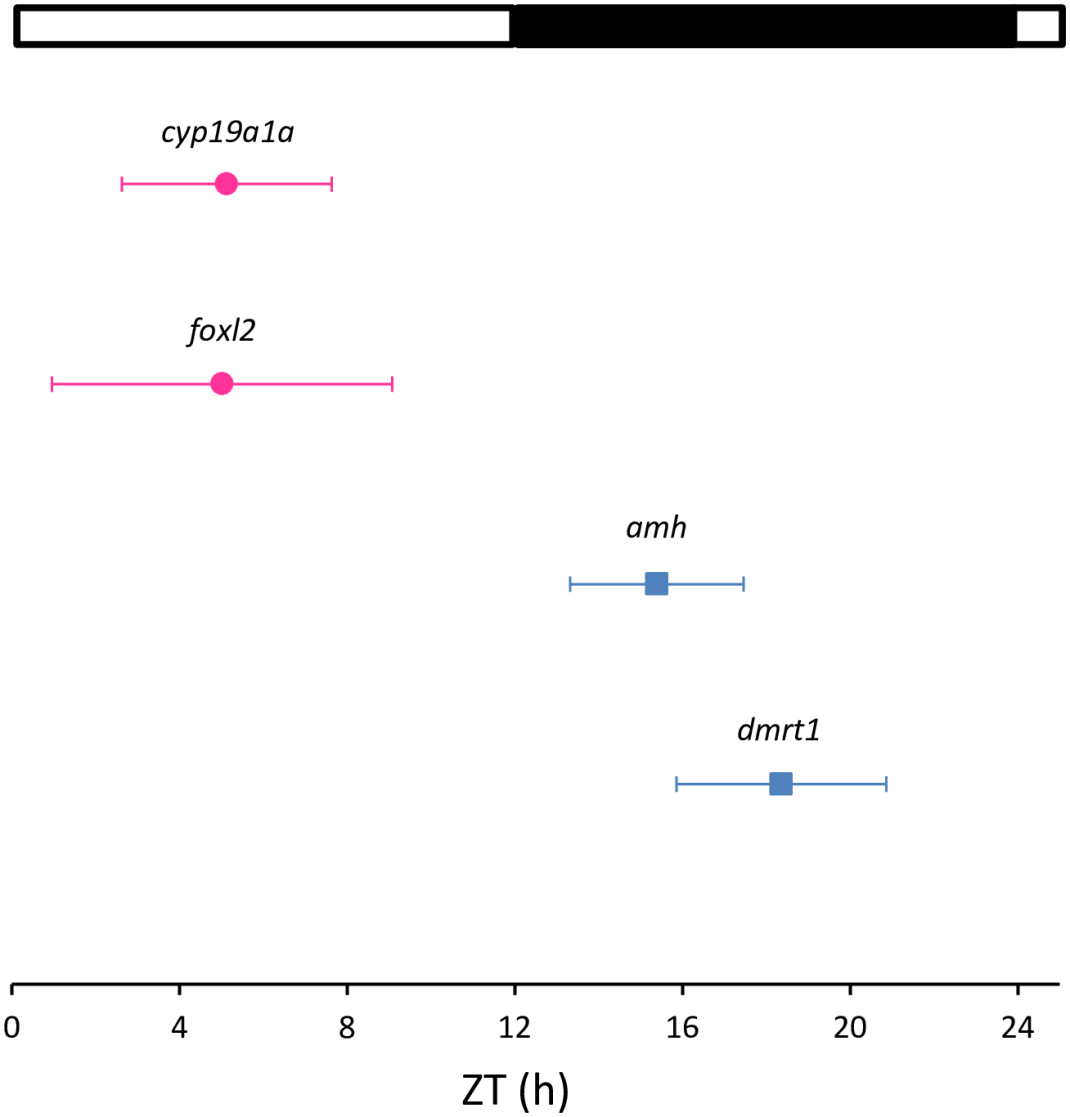
Map of acrophases. Only the genes that displayed a statistically significant daily rhythm (Cosinor, p<0.05) are included in the graph. The acrophase is indicated by symbols and the fiducial limits (set at 95%) are indicated by lateral bars. The pink circles and blue squares indicate the genes analyzed in the ovary and testes, respectively. The white and black bars above the graph represent the light and dark phase, respectively.

All the analyzed genes showed either daily rhythmicity (Cosinor, p<0.05) or significant differences among the time points (one-way ANOVA, p<0.05), or both. In females, *cyp19a1a* was expressed in the ovary and peaked in the middle of the light phase (ML, ZT 6 h) (one-way ANOVA p<0.001; Fig 2A). The analysis of this gene also revealed a sinusoidal rhythmic pattern with the acrophase at ZT 5:13 h (Cosinor, p<0.003; Table 2, Fig 3). No differences were observed in *foxl2* expression (as assessed by ANOVA), but sinusoidal rhythmicity was shown with the acrophase at ZT 5:02 h (Cosinor, p<0.021, Fig 2B, Table 2), which approached the achrophase of *cyp19a1a*.

**Table 2:** Mesor and amplitude values, given as relative expressions (r.e.), calculated by the Cosinor analysis. The degree of adjustment of the experimental data to the cosine function calculated by Cosinor is given by the percentage of variance. Values are indicated only for the parameters that showed a significant daily rhythm (Cosinor, p<0.05). NS indicates the genes that show no statistically significant rhythmicity (Cosinor, p>0.05), and a dash denotes the genes that were not analyzed.

		Female			Male		
Gene	Tissue	Mesor (r.e)	Amplitude (r.e.)	Variance (%)	Mesor (r.e)	Amplitude (r.e.)	Variance (%)
***cyp19a1a***	gonad	30.82	50.16	48.67	-	-	-
***foxl2***	gonad	8.19	5.23	18.16	-	-	-
***amh***	gonad	-	-	-	2367	3405	54.62
***cyp11b***	gonad	-	-	-	ns	ns	ns
***dmrt1***	gonad	-	-	-	130.82	151.64	42.18
***cyp19a1b***	brain	ns	ns	ns	ns	ns	ns
***cyp11b***	brain	ns	ns	ns	ns	ns	ns

**Fig 4:**
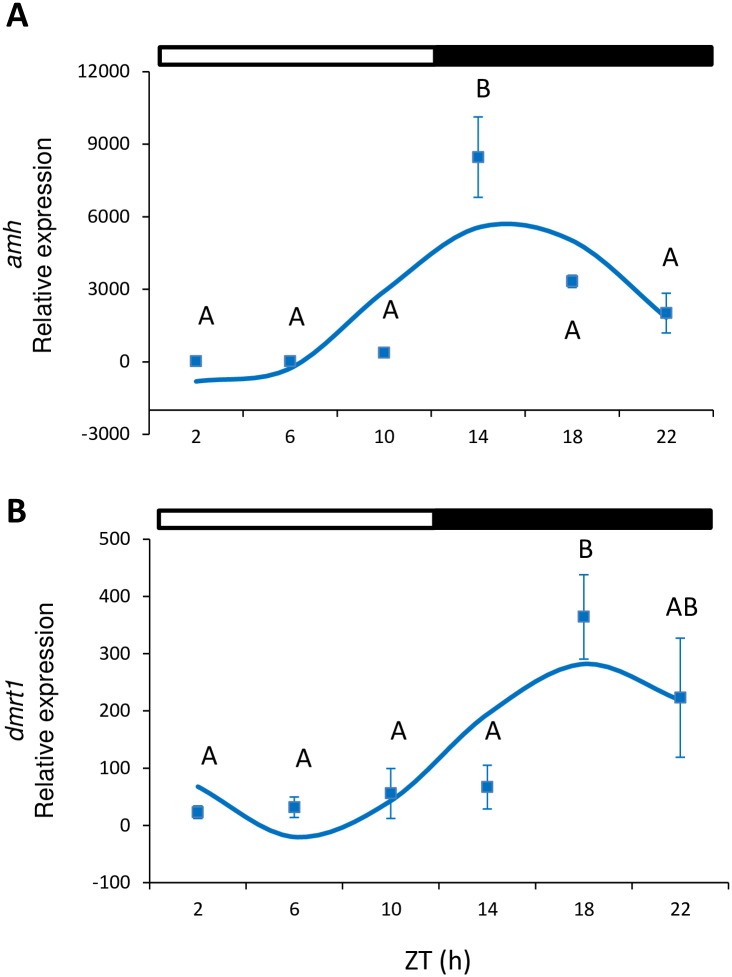
Relative gene expression of *amh* (A) and *dmrt1* (B) in testes. Squares indicate the relative gene expression (mean±SEM) at each sampled time point. Different capital letters indicate the statistically significant differences between time points (one-way ANOVA, p<0.05). The sinusoidal curve calculated by Cosinor (p<0.05) is indicated by the continuous line. The white and black bars above the graphs represent the light and dark phase, respectively.

In males, *amh* expression showed a significant peak of expression in testes at the beginning of the dark phase (ZT 14 h) (one-way ANOVA, p<0.001; Fig 4A). In addition, *amh* presented a significant sinusoidal rhythm, with the acrophase located at ZT 15:39 h (Cosinor, p<0.05; Table 2, Fig 3). *Dmrt1* displayed significant differences during the ZT (one-way ANOVA, p<0.005, Fig 4B), peaked at ZT 18 h and also showed a sinusoidal rhythmic pattern with acrophase at ZT 18:36 h (Cosinor, p<0.002, Table 2). *Cyp11b* expression was investigated in testes but displayed no significant differences, and either depended on the time of day (one-way ANOVA, p<0.05, Tukey’s *post hoc*, p = 0.053) or sinusoidal rhythmicity (Table 2).

**Fig 5:**
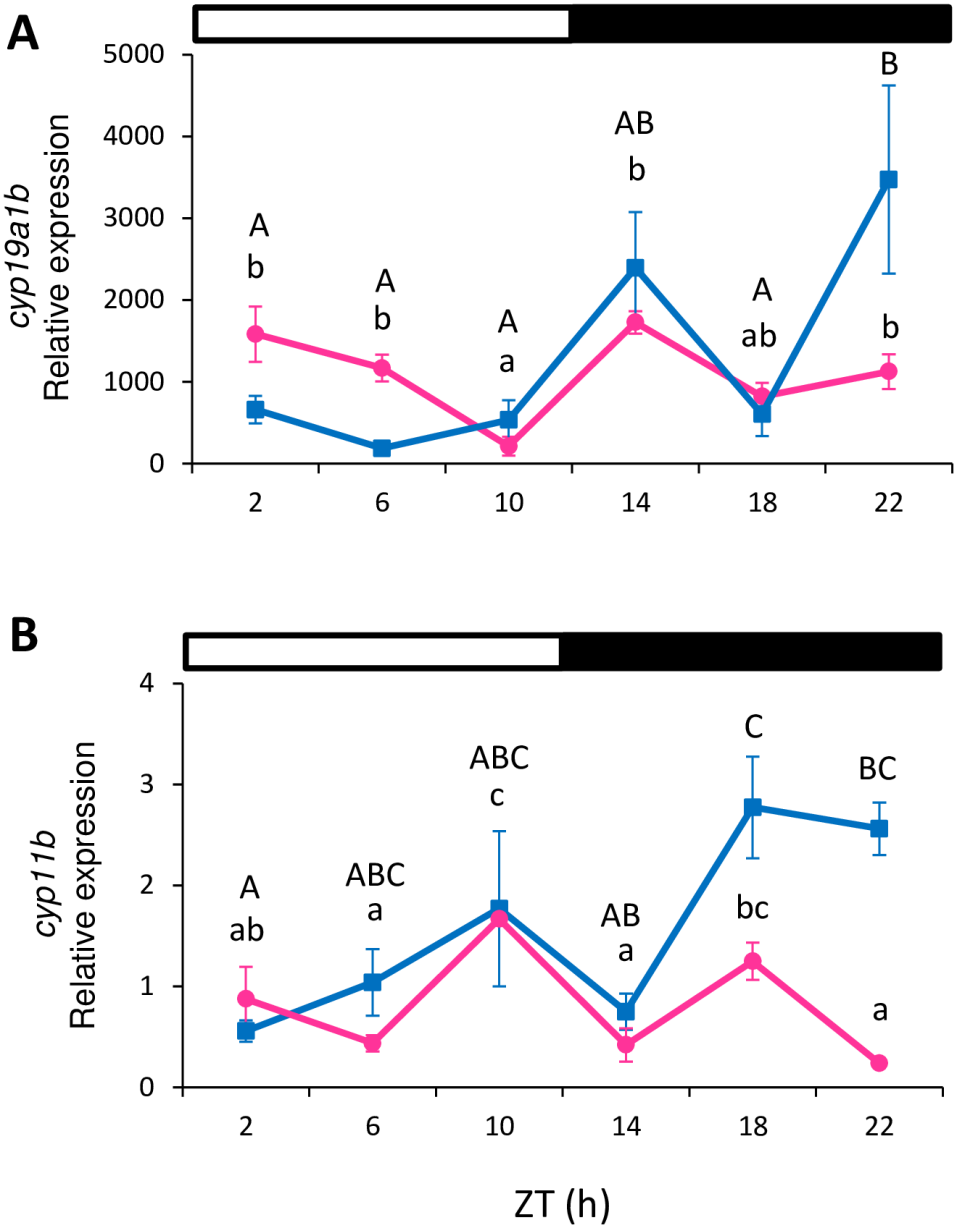
Relative gene expression of *cyp19a1b* (A) and *cyp11b* (B) in the brain. Brain samples were analyzed separately for females (pink circles) and males (blue squares). Data are expressed as mean±SEM. Different letters indicate the statistically significant differences between time points in the female (lower case letters) or male (capital letters) brains (one-way ANOVA, p<0.05) within each gene. The white and black bars above the graphs represent the light and dark phase, respectively.

Regarding *cyp19a1b* expression, in the brain both females and males showed statistically significant differences depending on the time of day. In males, the peak occurred in the dark phase (ZT 22 h), whereas values remained constant and the only decrease in expression was detected at ZT 10 h in females (one-way ANOVA, p<0.005; Fig 5A). *Cyp11b* expression presented time-dependent differences in both males and females. The highest expression in females occurred at ZT 10 h, immediately before light offset (one-way ANOVA, p<0.0001; Fig 5B), but occurred at ZT 18 h (in the middle of the night; MD) in males (one-way ANOVA, p<0.006; Fig 5B). For *cyp19a1b* and *cyp11b*, no significant daily rhythms were detected (Cosinor, p>0.05; Table 2).

## Discussion

Although the characterization, localization and expression of the key genes involved in steroidogenesis and reproduction have been extensively studied in many fish species, their daily rhythms have been poorly explored to date. The present findings revealed that the expression profiles of these genes are not flat, but change during a 24 h cycle. In the ovary, analyzed genes *cyp19a1a* and *foxl2* oscillated with a similar phase. The same occurred in the testis for *amh* and *dmrt1* expressions. However, the phases of these genes were opposite between sexes, as ovarian *cyp19a1a* and *foxl2* presented diurnal peaks, whereas *amh* and *dmrt1* in testes displayed nocturnal peaks.

Estrogens are essential hormones for fish reproduction, and aromatase is the key enzyme involved in their conversion from androgens [37, 44]. *Cyp19a1a* is present only in the ovaries of females and its expression follows a rhythmic pattern. Gonadal aromatase presented its maximum expression level in the light phase at ML and showed a diurnal pattern in phase with the other genes investigated in female gonad *foxl2*. However, *cyp19a1a* expression in the ovary did not occur in phase with *amh* expression in testes, which peaked at night. In contrast, brain *cyp19a1b*, the “neural aromatase”, presented similar profiles in both sexes. It should be noted that in previous studies *cyp19a1b* displayed the same level range of expression in both females and males [45–46]. While the role of aromatase in gonads has been largely studied, many questions about its role in the brain remain to be answered. There is evidence that aromatase plays a role in the control of reproduction by the brain [47], and displays different activity depending on the sex and life stage. In 1-year-old European sea bass (*Dicentrachus labrax*), brain aromatase activity was higher in premature males than in females and imnmature fish [48]. Seasonal variations in brain aromatase have also been detected, with maximum values observed during the spawning season [48].

Previous studies on steroid rhythmic levels in other fish species (e.g. *S*. *senegalensis*) have revealed that testosterone (T) and estradiol (E_2_) are both secreted mostly in a rhythmic manner [7]. According to Bayarri et al. [49] in European sea bass, plasma T in males, plasma and pituitary LH, and pituitary GnRH display daily rhythms with different acrophases. In snapper (*Pagrus auratus*), plasma E_2_ and T change during the day [9]. Japanese whiting *(Sillago japonica*) also presents a diurnal pattern of plasma E_2_ [50]. Hormonal rhythmicity is connected directly to rhythms of physiological and behavioral processes, such as reproduction and spawning, which usually coincide with the phase of the LD cycle that displays the greatest locomotor activity. In gilthead sea bream, spawning starts in the afternoon and continues for a few hours after night falls [51]. In Senegalese sole, spawning takes place when night falls and correlates with its nocturnal locomotor activity [7]. In sole, the daily peaks observed in T and E_2_ precede the time of spawning by a few hours [7, 52]. The European sea bass spawns at nighttime [53], which is preceded by a peak in plasma LH observed at dusk [49]. However, T and GnRH do not correlate with spawning, and peak when night ends and during the day, respectively [49]. In zebrafish, spawning takes place at dawn [29]. Ovulation in zebrafish shows a daily rhythm with the acrophase and coincides with spawning: end of darkness and beginning of the light phase [54]. Pituitary *fsh* and *lh* [30], and *amh* and *dmrt1* expression (Fig 4), peak at nighttime and precede spawning by several hours, while ovarian *cyp19a1a* and *foxl2* peak several hours after spawning (Fig 2). Although all these results illustrate a complex mechanism of regulation, it is important to emphasize that they highlight that the HPG axis of fish presents daily rhythms at all its levels. These rhythms on the HPG axis seem to be coordinated to lead to the timing of the spawning event, which occurs at the time of day at which success is higher, especially for spawn survival.

In fish, water temperature strongly influences the processes related with reproduction, such as sex differentiation [21, 55–57]. In zebrafish, water temperature has been reported to affect the sex ratio in early life stages [58]. It is most interestingly that daily thermocycles also affect sex differentiation compared with constant water temperatures [24]. Thus the sex ratio is affected not only by temperature itself, but also by the time of day at which temperature rises. These findings suggest the existence of a window of sensitivity at specific times of the day. The present results supported this hypothesis since both *cyp19a1a* and *amh* displayed daily rhythms with acrophases at different times of day. These findings indicate the complex mechanisms by which temperature may induce these sex ratio changes.

In summary, our results revealed, for the first time, the importance of time of day on the daily expression of the genes involved in reproduction, which should be considered when investigating sex steroid production and fluctuations of gene expression over a 24-hour cycle.
